# Salivary Cortisol Levels after Hydrotherapy and Land-Based Therapy as a Marker of Stress in Children with Psychomotor Developmental Disorders: A Pilot Study

**DOI:** 10.3390/jcm13144147

**Published:** 2024-07-16

**Authors:** María José Aguilar-Cordero, Sabina Michel-Araya, Jessica Pamela Noack Segovia, Julio Latorre-García, Ana María Rojas-Carvajal, Rafael Fernández Castillos

**Affiliations:** 1 CTS-367, Andalusian Plan for Research, Development and Innovation, University of Granada, 18071 Granada, Spain; mariajoseaguilarugr@gmail.com (M.J.A.-C.); sabi.michel.a@gmail.com (S.M.-A.); jnoack@santotomas.cl (J.P.N.S.); juliolatorrefisio@gmail.com (J.L.-G.); rafaelfernandez@ugr.es (R.F.C.); 2 Department of Nursing, Faculty of Health Sciences, University of Granada, 18071 Granada, Spain; 3 San Cecilio University Hospital (PTS), 18071 Granada, Spain; 4 Department of Nursing, University of Santo Tomás, Talca 3460000, Chile; 5 Neurotraumatology and Rehabilitation, Virgen de las Nieves University Hospital, 18071 Granada, Spain; 6 Ibs.Granada, Health Research Institute, 18071 Granada, Spain

**Keywords:** salivary cortisol, infants/children, physical therapy modes, hydrotherapy, land-based therapy

## Abstract

**Background:** The number of children experiencing postnatal situations of neurological risk (such as psycho-motor developmental disorders and delays) after birth has increased in recent years. These infants often require multiple pediatric interventions to address functional problems that might generate stress, anxiety, and discomfort. The aim of the present study is to determine whether the level of salivary cortisol, as a stress marker, increases after hydrotherapy and land-based therapy in children at risk of or currently presenting delayed psycho-motor development. **Methods:** Saliva samples were collected from 25 children (aged 3–36 months) between June 2022 and January 2023 at the Rehabilitation and Physical Medicine Clinical Management Unit of the Virgen de las Nieves University Hospital, Granada, Spain. Three samples were collected from each child, representing baseline, post-hydrotherapy and post-land-based therapy. **Result:** All salivary cortisol levels were within the normal range. Resting values were the highest, and both modes of therapy decreased salivary cortisol levels. There were no statistically significant differences between the two therapies. **Conclusions:** Both therapies appear to be useful for treating children with psychomotor developmental disorders without increasing stress during physiotherapy sessions. Although cortisol levels were slightly higher with hydrotherapy than with land-based therapy, this may be due to the small sample size.

## 1. Introduction

Improved rates of perinatal survival have led to growing numbers of children presenting with functional mobility problems, disabilities, and psychomotor developmental disorders. This is especially the case among preterm infants, of whom 50% present such disabilities [1,2,3,4]. To address this situation, infants may be admitted to pediatric rehabilitation programs in our service, based on hydrotherapy (HYDRO) and/or land-based therapy (LBT) techniques to normalize motor development patterns, in areas such as muscle tone, coordination, and balance. Moreover, this approach can enhance sensory integration, thus facilitating psycho-affective and language development [5,6]. However, it has been observed that many of the infants in the rehabilitation program show discomfort, unpleasantness, or crying during the therapies, especially LBT. This is supported by the literature, where frequent health check-ups, treatment, and use of some rehabilitation methods, may expose the patients to greater stress [3,7]. These reactions are not so common during HYDRO, which they seem to enjoy more, possibly due to the relaxing properties of water and the sensory and psychological effects produced [8,9,10]. Although these two therapy modes are frequently related, there is scant evidence as to whether one is better than the other for children suffering from pathologies arising in the perinatal period, or whether they impact stress levels [8,10,11,12]. As both HYDRO and LBT are usually provided as long-term therapies, they may produce visible effects on neurodevelopment in the first years of life [13], in addition to influencing general well-being. However, when a poorly tolerated therapy generates stress, natural defense mechanisms are activated. The consequent action of the cortisol hormone [1,14], which adjusts the stress response, generates energy, increases blood pressure, and may change both mood and behavior to defend against perceived threats [15]. A chronic imbalance in the level of this hormone alters the natural immune response, hinders healing, produces an over-expression of other hormones, facilitates obesity, and may damage the hippocampal cells, thus altering the development of cognitive processes and learning [11,13,16]. Therefore, levels of stress and cortisol need to be monitored, especially when long-term treatments are provided that can modify patterns of cortisol secretion. Furthermore, it is important to consider many other factors that may affect the cycle of cortisol release during the day, such as the type of social-affective attachment with caregivers, sleep quality, feeding schedules, level of physical activity, gestational age, and birth weight [17,18,19,20]. An objective means of carrying out this assessment without requiring an invasive procedure is to measure salivary cortisol levels (µg/dL). This physiological stress biomarker is a useful non-invasive pediatric tool [21,22] that enables the physician to indirectly assess the activity of the hypothalamic–pituitary–adrenal axis (HPA) [1,14]. For example, salivary cortisol studies have been conducted in children with central coordination disorders undergoing Vojta Therapy [3], similar to what was designed in this study. Studies have also been conducted in premature infants undergoing hydrokinesiotherapy in an intensive care unit [11], and in cardiovascular, motor, and combined several-day programs of early care therapy and kinesthetic tactile stimulation [12,18,23,24]. To date, no study has evaluated cortisol levels following a HYDRO and LBT intervention session in this specific population. This study will allow us to expand the existing knowledge base on the use of salivary cortisol in children undergoing physiotherapy. The study will also enable us to compare the emotional responses of children to the different physiotherapy options available and to identify alternative approaches for each case while respecting the individual needs of each child. In view of these considerations, the aim of this study is to measure salivary cortisol concentration as a marker of stress following LBT and HYDRO interventions, addressing the following research question: Do stress levels (salivary cortisol) increase after HYDRO and LBT in children at neurological risk?

## 2. Materials and Methods 

### 2.1. Design and Participants

**Figure 1:**
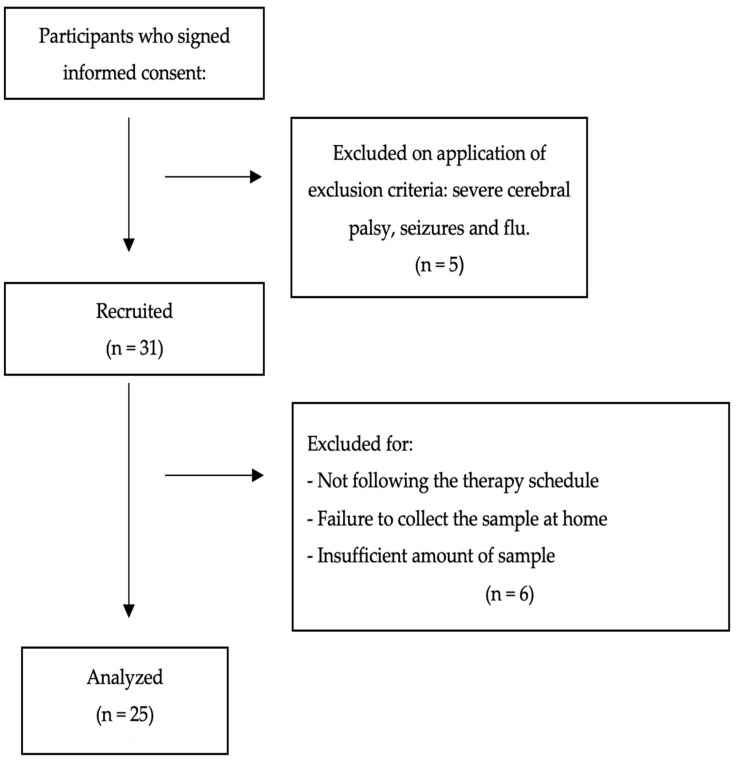
Flow chart.

The sample size was calculated from the number of pediatric rehabilitation patients seen monthly at the Virgen de las Nieves University Hospital in Granada. According to the data provided by the hospital, 115 children were seen in the pediatric rehabilitation service over a period of nine months. Therefore, it is estimated that 153 children would be seen in one year. Of these children, 80% attended HYDRO and LBT. Assuming a universe of 122 children with a margin of error of 10% and a confidence interval of 95%, we calculated that 55 children needed to be recruited. Initially, 36 were identified and their parents/guardians signed the informed consent form, but only 25 met the inclusion criteria and were finally included in our analysis (the selection protocol is detailed and illustrated in Figure 1).

The inclusion criteria applied were that the children should be aged 3–36 months, present mild-to-moderate psychomotor developmental delay, and provide an accurate sample. In addition, signed informed consent to participate was required in every case.

The exclusion criteria were the presence of severe neurological disease, severe behavioral disorder, autism spectrum disorder level 3, the systemic administration of corticosteroids, orthopedic surgery during the last three months, comorbidities, malnutrition, and maternal drug use during pregnancy. Information was collected through an informal interview on birth data, drugs used by mother and child, sleep habits of the child, and parental habits and illnesses to help determine the criteria for inclusion and exclusion from the study.

### 2.2. Ethical Approval

The study was approved by the Bioethics Committee of Biomedical Research of Granada Province, San Cecilio University Hospital (PTS), Junta de Andalucía (CEIM/CEI GRANADA, Approval No 2e70ffe4e6e114160a7369d336c8f4b0e6a303fc, 13 June 2022). All caregivers were informed about procedures, participated voluntarily, and signed the informed consent and data confidentiality form before entering the study.

### 2.3. Sample Collection and Transport to the Laboratory

Three saliva samples were collected from each child, one post-HYDRO, one post-LBT, and one at rest. The three samples were each obtained at the same chronological time (to synchronize each child’s circadian cortisol cycle with the level of cortisol excretion at the same time of day) but on different days within a period of three weeks. 

To ensure that salivary cortisol levels were not influenced by the new situations experienced, one week was allowed for adaptation to each of the physiotherapy interventions before sampling took place.

In assessing how the children responded to their LBT and HYDRO sessions, no new activities or exercises were introduced, and in every case, the treatment was provided by the same experienced physiotherapist, who has 10 years of experience with pediatric patients in both, LBT and HYDRO.

Saliva collection took place 25 min after the physiotherapy interventions [25,26,27,28], always (for LBT) in the same physiotherapy room or (for HYDRO) in the pool area, with the child in the mother’s arms. The collection was performed by another physiotherapist, who was previously present as an observer at each of the HYDRO and LBT sessions. In this way, the children were familiarized with the physiotherapist who collected the samples.

The children attended the HYDRO and LBT appointments according to a schedule coordinated with the physiotherapist and the research team. Samples were taken between 8:00 and 14:30 in every case. 

In the first (recruitment) stage of the study, the children’s parents/guardians were given written and verbal information about the proposed participation in the study. If the inclusion and exclusion criteria were met, the informed consent form was signed. 

**Figure 2:**
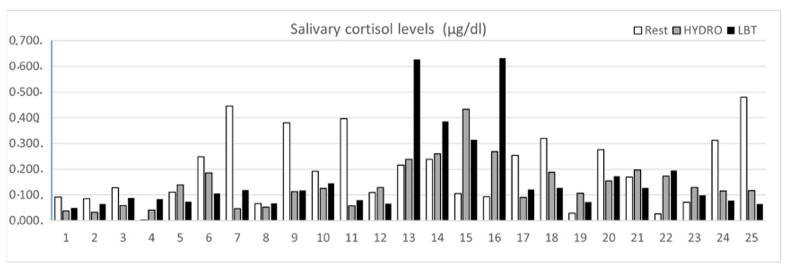
Salivary cortisol concentration values at rest, post-HYDRO, and post-LBT collected in each of the 25 children. On the *X*-axis, for each child, three bars are represented. White bars represent resting salivary cortisol values; grey bars, the post-HYDRO salivary cortisol values; and black bars, the post-LBT salivary cortisol values. The *Y*-axis represents cortisol values in micrograms per deciliter (µg/dL).

The second stage was devoted to sample collection. To collect each of the three samples, the investigator first ensured that the child had not had a febrile episode in the past 48 h and had not eaten for at least one hour before collecting the saliva. If these two criteria were met, the sample was collected according to the following protocol (Figure 2):

Phase 1: The caregiver was instructed to arrive at the scheduled time, to perform a 30 min HYDRO session, and then to wait for about 25 min near the pool in an area with a suitable temperature and conditions of comfort for the child. The post-HYDRO saliva sample was then collected. 

On the same day, the date was established for the next physiotherapy appointment, to coincide with the post-HYDRO saliva sample collection.

Phase 2: In the land-based physiotherapy session, the same structure was followed. The caregiver was instructed to arrive at the appointed time, to complete a 30 min LBT session, and then to wait for approximately 25 min near the therapy room. During this waiting period, the caregiver was given verbal and written information on how to collect, preserve, and transport the saliva sample to be collected in Phase 3.

Phase 3: The procedure for collecting the resting sample was agreed upon and coordinated with the caregivers. This sample was collected at the family home, at a time when there were no scheduled therapy sessions, medical procedures, or activities that might alter the basal cortisol parameters. The child should remain at relative rest for 30 min prior to sampling. The caregivers were instructed to collect the sample at the same time of day as in the previous collections. After extraction, the sample was to be stored in a normal refrigerator at 2–4 °C until it was transported, properly preserved, and given to the physiotherapist in charge of collecting the samples, who delivered it to the laboratory within 72 h [29].

All participants were recruited and all HYDRO and LBT sessions were performed at the Virgen de las Nieves University Hospital, but the samples were analyzed in the laboratory of San Cecilio University Hospital (PTS). In total, data were collected from 25 children, who provided 75 samples of saliva.

### 2.4. Sample Analysis Procedure

Saliva samples were collected from healthy children using a sterile Salivette^®^ collection kit as follows (SARSTEDT AG & Co. KG, Nümbrecht, Germany). The cotton swab was held in the sublingual and cheek area of each infant’s mouth for 2–3 min. The tubes containing the swabs were transported during the same week to the laboratory, where salivary cortisol concentrations were determined by liquid chromatography, based on the tandem mass spectrometry method (LC-MS/MS). After transport to the laboratory, the samples were processed the same day or preserved at 5 °C, and analyzed within the same week. Cortisol remains stable in saliva samples at room temperature, and it does not degrade for about a month (10%) at room temperature [30], and it gives the possibility of several freezing cycles [23,29].

The assumed normal values for minimum and maximum cortisol levels in children in the studied age range were based on the PTS laboratory reference values (0.11–0.76 µg/dL at 8 a.m. and 0.015–0.100 µg/dL at 11 p.m.). 

### 2.5. Physiotherapy Interventions

#### 2.5.1. Hydrotherapy

The weekly individualized hydrotherapy sessions were each performed for 30 min, with a variety of activities designed to develop skills appropriate to the child’s age. These included adaptation to the environment, tolerating water on the face, and adapting vision during immersion through motor games. Proprioceptive maneuvers such as passive mobilization of the upper and lower limbs, trunk control in prone, supine, and sitting positions, modeling techniques to develop trunk rotation, quadruped position, underwater propulsion, vestibular exercises to improve balance, facilitation of frontal displacement during immersion, rolling, standing, walking and sensory stimulation through the movement in the water and its temperature [9,31]. The pool area was comfortable and warm at all times. The characteristics of the pool water were suitable for babies, with a temperature of 34–36 °C, chlorine content of 0.5 ppm, saline purification, and pH 7.6. The temperature of the environment is maintained at 26–27 degrees Celsius, which is considered a temperate climate.

#### 2.5.2. Land-Based Therapy

The LBT sessions were also weekly and individualized, with a duration of 30 min, involving proprioceptive maneuvers as passive and active mobilization of the upper and lower limbs, facilitation techniques to develop head and trunk control in prone, supine, sitting, and quadruped positions, motor games, vestibular and modeling strategies to achieve trunk rotation, promotion of crawling, balance, rolling, standing position, gait and sensory stimulation activities. These activities were the same as those performed in the water but in a different environment. The same pediatric physical therapist conducted the sessions in every case. These routines are standard procedure in the hospital. No specific therapies were designed for the study participants because the study aim was to determine whether this form of therapy influenced levels of salivary cortisol and hence stress.

### 2.6. Statistical Analysis

Each of the demographic characteristics of the study population is described by the corresponding median, standard deviation, and percentage (Table 1). In addition, a descriptive chart highlights individual changes in salivary cortisol levels (Figure 2). The descriptive group statistical analysis summarized in Table 2 shows the median and standard deviation values obtained for the quantitative outcomes. Baseline cortisol levels were measured in each patient to reveal the effects produced by HYDRO and LBT in this respect. The effects were compared using paired samples, comparing each patient’s baseline results with those of the interventions. The non-parametric Wilcoxon test was performed, due to the small number of cases considered. The statistical analysis was conducted using the SPSS 22 program.

## 3. Results

### 3.1. Demographic Information

The general descriptive characteristics of the participants are described in Table 1. Regarding the birth history of these infants, 44% were preterm and almost 50% were female. The mean gestational age was 35 weeks (±5.67) and the mean birth weight was 2496 g (±1022.59). Regarding the diagnoses, four presented alterations in several areas of psychomotor development, such as cerebral palsy or specific syndromes, producing global developmental delay (GDD). The other infants considered at neurological risk presented mild alterations or delays in only one area (sensory, motor deficits such as plagiocephaly, dolichocephaly, cervical torticollis, muscle contractures, alterations in muscle tone or prematurity), suggesting they should be included in the infant rehabilitation program for treatment and observation. The diagnoses were FOXG1 syndrome, Cri du Chat syndrome, Van der Woude syndrome, dysmorphic syndrome, and achondroplasia. Pre-, peri-, and post-natal complications such as hypotonic syndrome, spastic tetraparesis, mild hypoxic-ischemic encephalopathy, mild intraventricular hemorrhage, paroxysmal non-epileptic disorder, hyperextension of the neck, torticollis, plagiocephaly, dacryocele and iris coloboma, jaundice (4 months), bronchopulmonary dysplasia, respiratory distress, chorioamnionitis, oligohydramnios, and twin pregnancy.

### 3.2. Salivary Cortisol Levels after HYDRO and LBT

Figure 2 shows the cortisol levels recorded in each of the 25 children included in the analysis. The white bars represent the values at rest; the gray bars are those obtained after HYDRO, and the black bars are those obtained after LBT. The *Y*-axis represents the cortisol values, in micrograms per deciliter.

In most cases, the resting values were significantly higher than those obtained after physiotherapy. Although outlier cases with extreme values were observed in cases 13 to 16, these values were within normal laboratory reference limits.

### 3.3. Statistical Group Analysis

In the box and whisker diagram, the first box plot represents the resting salivary cortisol values, with values that are higher and wider, with a minimum of 0.002 (µg/dL), maximum of 0.480 (µg/dL) and median of 0.169 (µg/dL). 

The second box plot represents the values analyzed after the HYDRO session, with a minimum value of 0.033 (µg/dL), a maximum of 0.433 (µg/dL), and a median of 0.125 (µg/dL).

Finally, the third box plot shows the salivary cortisol values obtained after the LBT session, with a minimum value of 0.050 (µg/dL), a maximum of 0.632 (µg/dL), and a median of 0.106 (µg/dL).

Table 2 shows the values determined in the paired samples analysis and the non-parametric test, revealing a notable decrease in salivary cortisol levels after each of the interventions, compared to the baseline values. However, there were no statistically significant differences between the interventions.

## 4. Discussion

**Figure 3:**
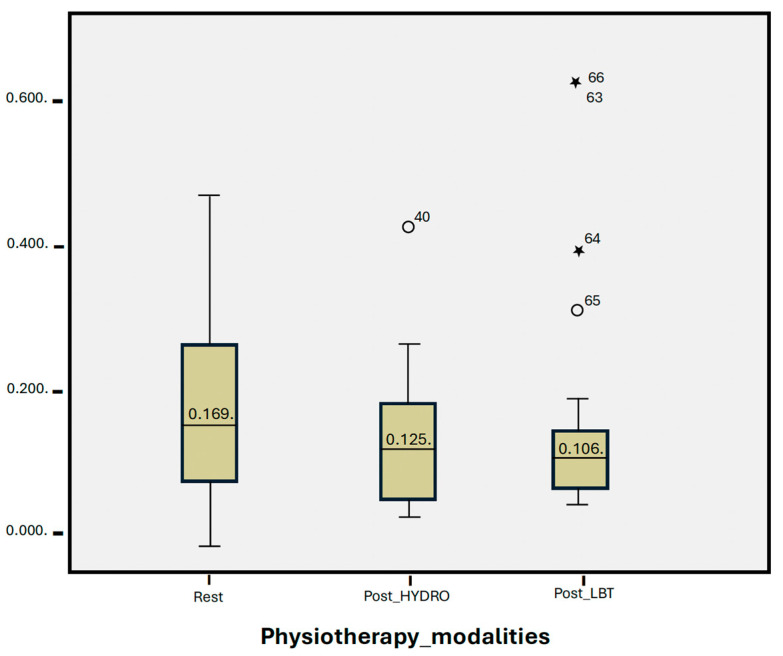
Group statistics analysis, box and whisker diagram. The *Y*-axis represents salivary cortisol values in micrograms per deciliter (µg/dL). The *X*-axis shows 3 conditions: resting salivary cortisol values, post-HYDRO salivary cortisol values, and post-LBT salivary cortisol values. The asterisks represent outliers.

Our analysis shows that both HYDRO and LBT show a trend towards a reduction in salivary cortisol, despite not significantly lowering levels (Figure 3). In our study, cortisol levels were slightly higher after HYDRO than after LBT, but the difference was not statistically significant. However, several other studies have reported that HYDRO more effectively reduces cortisol levels. In 2016, Oliveira-Tobinaga et al. [11] evaluated the effects of hydrokinesiotherapy in 15 premature newborns treated in a NICU, finding a statistically significant reduction with respect to preintervention cortisol levels, from 0.41 ± 0. 14 µg/dL to 0.29 ± 0.09 µg/dL (*p* = 0.004). In 2018, Freitas et al. [23] conducted a similar study, but in this case, the therapy session did not involve movement in the water, only immersion. These authors observed a tendency for cortisol levels to increase, but the difference was not statistically significant. Also in 2018, Shaw et al. [32], evaluated stress levels in preterm newborns after an assisted physical exercise program, finding a significant reduction in salivary cortisol of 0.08 µg/dL (95% CI: −0.16 to −0.002; *p* = 0.04) at 30 min post-exercise, compared to the value at rest. No differences were found at 90 and 120 min post-exercise, compared to the values at rest. These findings suggest that exercise therapy does not increase levels of stress in the child. In addition, improvements were observed in physiological parameters, mineral content, and bone growth levels. In 2021, Durán-Carabalí et al. [33] assessed stress via salivary cortisol and alpha-amylase in 41 children aged 6–86 months with cerebral palsy (CP) who underwent a neurodevelopmental treatment program. These authors recorded higher baseline cortisol values in the CP group than in the control group (healthy children). However, cortisol levels at 20 min post-intervention were significantly lower than at baseline and at 5 min post-intervention. The participation of caregivers in performing the therapies had no significant effects on cortisol levels. In 2021, Kiebzak et al. [3] evaluated cortisol levels after Vojta therapy and measured an immediate post-intervention salivary cortisol increase to 12.5 nmol/L. However, at 20 min post Vojta therapy, the levels had decreased significantly, to 9.5 nmol/L, but remained above the pre-intervention values. In 2009, White-Traut et al. [34], studied stress reactivity via pre-intervention, immediate post-intervention, and 10 min post-intervention cortisol levels, comparing a tactile stimulation therapy (massage) with a multisensory stimulation therapy (massage plus other stimuli). These authors found statistically significant differences (*p* = 0.01), with slightly lower cortisol levels for multisensory therapy than for tactile stimulation alone. In 2021, Tai and Lau [35] assessed salivary cortisol levels after a kinesiological education program (brain gymnastics) in 37 children aged 3 to 6 years with special educational needs. The intervention was conducted once weekly, for an hour, over a total period of ten weeks, after which a decrease in cortisol levels was recorded. However, the results were not significant. Finally, in 2020, Efe et al. [36] reported a decrease in serum cortisol levels after an exercise program for very low-weight preterm newborns treated in a NICU. Bone mineral density levels were also enhanced.

These reports show mixed results but with a tendency for stress levels to decrease following intervention therapies. This suggests that movement, exercise, and appropriate multisensory stimulation contribute to reducing stress levels in babies, although outcomes may depend on the type of exercise performed. Thus, according to a meta-analysis conducted in 2015 [37], cortisol levels in saliva vary according to the type of exercise, the duration, and the intensity of the session. In 2019, Wegner et al. [18], studied the impact of a 10-week physiotherapy program in children and found that cardiovascular exercise raised levels of cortisol release, whereas motor exercises decreased the release of this hormone. On the other hand, Hill et al. [38] and Ntovas et al. [39] showed that low-intensity cardiovascular exercise (representing <40% maximum oxygen consumption) does not significantly raise circulating plasma cortisol levels. Nor does it provoke discomfort or activate stress mechanisms. Furthermore, it reduces cortisol levels in children. In our study, both interventions considered are low-intensity exercises focused on enhancing motor skills and providing multisensory stimulation. The only significant difference lies in the environment in which the exercise takes place. HYDRO is an excellent rehabilitation medium with a greater cardiovascular component than LBT, providing enhanced sensory stimulation throughout the body thanks to its movement-facilitating properties and relaxing, pain-relieving impact [40,41,42]. Compared to LBT, HYDRO offers a broader cardiovascular component, mobilizing more body systems at the same time, and thus producing greater exercise intensity. This could explain why cortisol levels were slightly higher in HYDRO. In addition, the parents reported that their children had better sleep quality and mood after the HYDRO sessions, helping regulate long-term cortisol levels despite the short-term increase. Overall, thus, the effects of HYDRO could be very beneficial in this respect.

It should be emphasized that cortisol levels vary greatly among individuals, and are affected by personality type, genetics, coping resources in the face of stress-producing stimuli [4], sleep quality [43,44,45], establishment of the circadian cortisol rhythm, its regulation and the HPA axis response. Moreover, pre-, peri-, and post-natal processes generate intra and inter-individual differences [46,47]. In 2017, Van Bodegom et al. [48] concluded that periods of stress in critical stages of growth and development can provoke long-term alterations in the HPA axis. Cortisol, as a product of this axis, easily crosses the blood–brain barrier, affecting its function and behavior. In 2021, Gao et al. [49] reported that changes in cortisol levels due to painful procedures experienced during the neonatal period might alter the neurobehavioral development of preterm newborns and continue producing anxiety in children, adolescents, and even adults. One of the milestones infants are expected to reach in motor, cognitive, and social development is that of sleeping through most of the night. This is achieved by 70–80% of children by the age of nine months. However, it may be hampered by anxiety, possibly related to the resistance offered to go to bed, difficulties at the onset of sleep, and problematic night awakenings (which are directly associated with reduced cortisol levels in the morning) [50]. In consequence, the ability to regulate cortisol levels is immature. These important issues will be considered in a forthcoming study. 

Stress assessment during growth processes is relevant to promoting development, learning, and quality of life. In 2016, Bourseul et al. [7] studied a group of children aged 8–15 years with CP who experienced difficulty in communication when they were physically manipulated in physiotherapy treatments. Of these children, 35–48% experienced pain and discomfort during their daily life activities in institutions and rehabilitation centers. Zhao et al. [12] also found that certain physiotherapeutic interventions caused pain. However, the therapies included in the latter analysis included head acupuncture, neuromuscular electrical stimulation, and traditional Chinese manipulation, all of which may be considered highly invasive. 

Salivary cortisol is considered a reliable biomarker of stress [21], but certain aspects of this indicator need to be considered. In 2019, Morera et al. [22] confirmed the usefulness of this tool, observing that it is non-invasive and that specimens are easy to transport and preserve. Moreover, it represents only 10% of the cortisol present in the blood, and quantification by LC/MS-MS is too costly for large-scale sampling. The reference values used in the Clinical Analysis Department of the San Cecilio University Hospital (PTS) are similar to those reported elsewhere [46] with a minimum morning value of 3.5 nmol/L (0.15 µg/dL) and a maximum of 27.8 (0.90 µg/dL, for infants aged 3–9 months. The saliva sample collection protocol applied in the present study is based on that described in the literature. As a practical, economical approach, and in conjunction with the Clinical Analysis Department of the PTS, we decided to collect the post-intervention and resting samples on the same schedule, according to the diurnal cycle of each child. This made the samples comparable and reduced the use of saliva collection tubes (to three per child), compared to other studies, which in most cases collected saliva in pre-post intervention sessions [3,11,12,23,24]. In our study, cortisol levels were measured at 20–30 min post-intervention, which seemed appropriate since maximum levels of circulating cortisol are released at this interval after a stressful event. On the other hand, in some extreme cases, the opposite was observed, i.e., resting cortisol levels were lower than in the post-intervention sample. In the four children in question, these extremes of salivary cortisol and stress may have occurred due to the application of strict feeding schedules, producing a lower degree of tolerance pre-feeding. One of these infants cried if not fed immediately after therapy, which coincided with the collection of the saliva sample. Another, with achondroplasia, had low sleep quality due to apnea. Another explanation for these differences in cortisol values could be the type of attachment between the baby and its parents [51], personality traits, and individual day- and night-time variations. Significantly, two of the mothers in question were smokers; research has shown that tobacco exposure and neuropsychological stress in children are directly related [52]. These four cases all concerned male children, assessed in the same month, born at term, and aged between 3 and 7 months.

### Strengths and Limitations

The Virgen de las Nieves University Hospital is the only public hospital in the city and its surrounding area that offers this type of aquatic therapy for infants with the characteristics we describe. The present study should be considered preliminary, as it was only possible to analyze a small population sample, due to the difficulty of coordinating schedules, the lack of time of the parents, limited human and financial resources for data collection, non-attendance at the therapies in some cases and the frequent impact of flu. Further analysis is required, with larger groups and a more homogeneous population, to better identify variables related to salivary cortisol levels, and to establish statistical significance in the results described. Future studies in this field should include assessments of sleep quality and mood, either after each session or after a period of therapy, and measure heart rate in order to determine the level of exercise intensity experienced during the sessions and to relate this to salivary cortisol levels.

## 5. Conclusions

The objective of this study was to determine and evaluate the variation in salivary cortisol levels as a measure of stress produced after HYDRO and LBT in children at risk or with disorders and delays in psychomotor development between 3 and 36 months of age. The results showed a trend towards a decrease in salivary cortisol levels after both therapies, in most cases with no counterproductive effects or increased stress. Overall, these multisensory therapies have a positive effect on stress levels in the population sample considered. Both therapies are highly recommended as physiotherapy techniques. In our study cohort, no significant differences were found between the two techniques. However, this analysis was based on a small sample of children with different diagnoses, and with inter- and intra-individual differences in cortisol levels, which makes it difficult to extrapolate from the results obtained. Therefore, a study with a larger sample size should be conducted to validate and extend our findings.

Nevertheless, this study enabled us to evaluate and guide the organization of rehabilitation therapies according to the personality and response of each child, whilst avoiding possibly counterproductive effects. Therapies should be carefully selected to support the child in all aspects of development. This is especially true for children with multiple medical problems.

## Figures and Tables

**Table 1:** Demographic characteristics.

Outcome	Description		N	%
Sex	Female		12	48
	Male		13	52
Gestational age, mean *(SD)			35.52	(5.67) weeks.
Premature			11	44
Age, mean (SD)			7.46	(7.54) months.
Birth weight, mean (SD)			2496	(1022.59) grams.
Functional diagnosis	Neurological risk		21	84
	GDD		4	16
COVID-19 infection			12	48
Nutrition	Breastfeeding		11	44
	Mixed		9	36
	Formula		5	20
Maternal age, mean (SD)			33.48	(5.14) years.
Parents’ educational level		Basic/high school	6	24
		University	19	76

*SD: standard deviation.

**Table 2:** Group analysis results.

	Median	* SD	*p* Value
**Resting**	0.169	0.13	
**Post-HYDRO**	0.125	0.09	0.107
**Post-LBT**	0.106	0.32	0.271

* Values correspond to Figure 3.

## Data Availability

All the data for the study are available from the authors on reasonable request.
